# Vitamin D_3_ Receptors and Metabolic Enzymes in Hen Reproductive Tissues

**DOI:** 10.3390/ijms242317074

**Published:** 2023-12-03

**Authors:** Anna Hrabia, Kinga Kamińska, Magdalena Socha, Małgorzata Grzesiak

**Affiliations:** 1 Department of Animal Physiology and Endocrinology, University of Agriculture in Krakow, Al. Mickiewicza 21, 31-120 Krakow, Poland; magdalena.socha@urk.edu.pl; 2 Doctoral School of Exact and Natural Sciences, Jagiellonian University, Gronostajowa 9, 30-387 Krakow, Poland; kinga.kaminska@doctoral.uj.edu.pl; 3 Department of Endocrinology, Institute of Zoology and Biomedical Research, Faculty of Biology, Jagiellonian University, Gronostajowa 9, 30-387 Krakow, Poland

**Keywords:** vitamin D_3_ receptors, VDR, PDIA3, vitamin D_3_ metabolism, 1α-hydroxylase, 24-hydroxylase, reproductive system, chicken

## Abstract

In recent years, vitamin D_3_ has been revealed as an important regulator of reproductive processes in humans and livestock; however, its role in the female reproductive system of poultry is poorly known. The aim of this study was to examine vitamin D_3_ receptor (VDR and PDIA3) and metabolic enzyme (1α-hydroxylase and 24-hydroxylase) mRNA transcript and protein abundances, and protein localization within the hen ovary, oviductal shell gland, pituitary, liver, and kidney. We demonstrated, for the first time, the patterns of the relative mRNA and protein abundances of examined molecules in the ovary, dependent on follicle development and the layer of follicle wall, as well as in other examined organs. Immunohistochemically, PDIA3, 1α-hydroxylase, and 24-hydroxylase are localized in follicular theca and granulosa layers, luminal epithelium and tubular glands of the shell gland, pituitary, liver, and kidney. These results indicate that reproductive tissues have both receptors, VDR, primarily involved in genomic action, and PDIA3, probably participating in the rapid, non-genomic effect of vitamin D_3_. The finding of 1α-hydroxylase and 24-hydroxylase expression indicates that the reproductive system of chickens has the potential for vitamin D_3_ synthesis and inactivation, and may suggest that locally produced vitamin D_3_ can be considered as a significant factor in the orchestration of ovarian and shell gland function in hens. These results provide a new insight into the potential mechanisms of vitamin D_3_ action and metabolism in the chicken ovary and oviduct.

## 1. Introduction

The proper function of female reproductive system in birds depends on the coordinated actions of pituitary gonadotropins, ovarian steroid hormones, and locally produced and acting paracrine/autocrine factors within the ovary and oviduct [1,2,3]. The ovary of the laying hen includes follicles at all stages of development, from primordial and primary (up to 1 mm in diameter) located in the ovarian stroma, through prerecruited follicles including white (1–4 mm; WF) and yellowish (4–8 mm; YF) follicles, to recruited yellow follicles arranged into preovulatory hierarchy (>8–36 mm; usually five to eight follicles; Fn–F1). The preovulatory hierarchy is kept quite constant due to the selection on a near-daily basis of one follicle from the group of 6–8 mm follicles [4]. The largest yellow follicle, referred to as F1, is the first to ovulate and then the next largest (F2). The oviduct, consisting five parts, i.e., the infundibulum, magnum, isthmus, shell gland, and vagina, is responsible for the egg formation and oviposition (for review see [3]).

Among multiple factors regulating the function of the female reproductive system, including birds, is vitamin D_3_ [5,6,7,8]. It is supplied through feed or produced endogenously from 7-dehydrocholesterol in the skin upon UVB irradiation [9]. The hormonally active form of vitamin D_3_ (1α,25-dihydroxyvitamin D_3_; calcitriol) is synthesized in the kidney by 1α-hydroxylase (*CYP27C1* gene in birds [10,11], *CYP27B1* gene in mammals) from 25-hydroxyvitamin D_3_ produced in the liver by 25-hydroxylase. Subsequently, active vitamin D_3_ is catabolized by 24-hydroxylase (*CYP24A1* gene) to inactive calcitroic acid [12,13,14,15]. Metabolism of vitamin D_3_ can occur not only in the liver and kidney, but also in other organs expressing metabolic enzymes, such as the female reproductive system [8,16]. Circulating concentrations of vitamin D_3_ are considerably affected by hormonal and environmental factors such as nutrition, season, light, and genetics [9].

Vitamin D_3_ has a variety of physiological roles. Besides the best known regulation of calcium uptake, it is an essential regulator of reproductive processes in humans and animals (for review see [8,16,17]). Insufficiency/deficiency of vitamin D_3_ contributes to serious reproductive disturbances [18,19]. For example, hens fed a vitamin D_3_-deficient diet frequently lay thin-shelled and soft-shelled eggs [18].

Vitamin D_3_ primarily exerts its effects through the classical nuclear vitamin D_3_ receptor (VDR) and subsequent downstream activation or suppression of gene expression [13] as well as via activation a rapid non-genomic pathways [20]. One of the proteins possibly responsible for a rapid response to vitamin D_3_ is the protein disulphide isomerase family A member 3 (PDIA3) [15,21], of which expression has been discovered in mammalian reproductive organs [22]. PDIA3 interacts with caveolin 1, the membrane protein of caveolae, and activates the non-genomic signaling pathways, including the phospholipase C, phospholipase A2, protein kinase C, and Wnt family member 5 pathways, as well as mitogen-activated protein kinases and phosphatidylinositol-3 kinase pathways (for more details see [8,21]). Although vitamin D_3_ is essential for maintaining eggshell quality and egg production in hens, the information concerning the expression of vitamin D_3_ receptors and local synthesis- and metabolism-related enzymes in the avian ovary and oviduct is scarce. So far, a vitamin D_3_ binding site or VDR transcripts and protein were demonstrated in the chicken ovary [23,24] and oviduct [25,26]. Suggested roles of vitamin D_3_ in the hen ovary were attributed to the regulation of cell proliferation, steroidogenesis, and expression of genes involved in follicle development [24,27,28]. In the hen oviduct, VDR was closely related to eggshell calcification [25]. In the present study, we examined the hypothesis that the chicken reproductive system is also a site of vitamin D_3_ non-genomic action and its metabolism. Accordingly, VDR and PDIA3 receptors, as well as vitamin D_3_ synthesizing (1α-hydroxylase) and catabolizing (24-hydroxylase) enzymes mRNA transcript and protein abundance, and localization were examined within the hen ovary in relation to follicle development and in the oviductal shell gland (place of eggshell formation). Additionally, mentioned molecules were investigated in the pituitary, liver, and kidney.

## 2. Results

### 2.1. Transcript Abundances of VDR, PDIA3, CYP27C1, and CYP24A1 mRNA in the Hen Reproductive System

**Figure 1:**
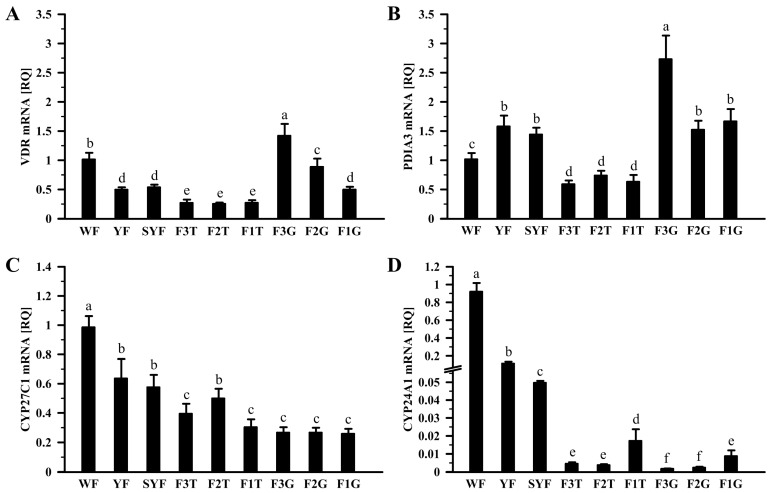
Relative mRNA transcript abundance of *VDR* (**A**), *PDIA3* (**B**), *CYP27C1* (**C**), and *CYP24A1* (**D**) in the hen ovary. Each value represents the mean relative quantity (RQ) ± standard error of the mean from 6 hens normalized to *18S rRNA* and standardized to the expression in the WF. Values marked with different letters differ significantly (*p* < 0.05; Kruskal–Wallis ANOVA on ranks, followed by the Student–Newman–Keuls test). Abbreviations: *VDR*, vitamin D_3_ receptor; *PDIA3*, protein disulfide isomerase family A member 3; *CYP27C1*, cytochrome P450 family 27 subfamily C member 1 = 1α-hydroxylase; *CYP24A1*, cytochrome P450 family 24 subfamily A member 1 = 24-hydroxylase; WF, white follicles, 1–4 mm in diameter; YF, yellowish follicles, 4–8 mm; SYF, small yellow follicles, 8–12 mm; F3-F1, tree of the largest yellow preovulatory follicles (26–35 mm, F3 < F2 < F1); G, granulosa layer; T, theca layer.

**Figure 2:**
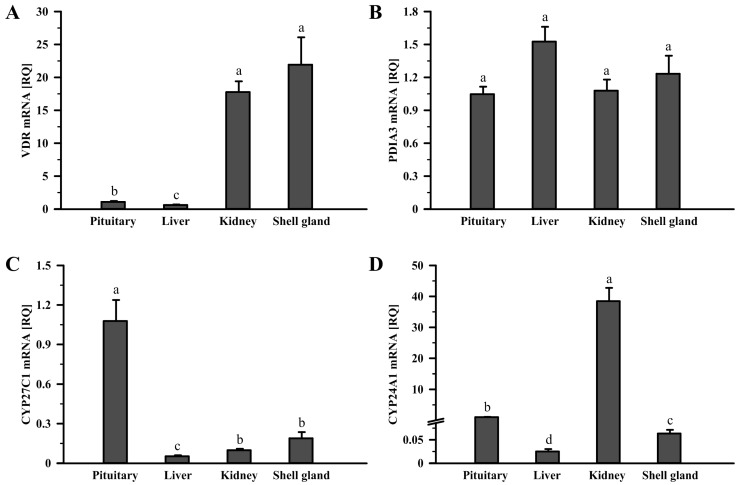
Relative mRNA transcript abundance of *VDR* (**A**), *PDIA3* (**B**), *CYP27C1* (**C**), and *CYP24A1* (**D**) in the hen pituitary, liver, kidney, and shell gland. Each value represents the mean relative quantity (RQ) ± standard error of the mean from 6 hens normalized to *18S rRNA* and standardized to the expression in the pituitary. Values marked with different letters differ significantly (*p* < 0.05; Kruskal–Wallis ANOVA on ranks, followed by the Student–Newman–Keuls test). Abbreviations as in Figure 1.

The relative abundances (RQ) of *VDR, PDIA3, CYP27C1,* and *CYP24A1* mRNA transcripts in the ovarian follicles during development, pituitary, liver, kidney, and shell gland are shown in Figure 1 and Figure 2. Within the ovary, the lowest *VDR* and *PDIA3* mRNA levels were in the theca layer of the largest preovulatory follicles and the highest in the granulosa layer of these follicles (*p* < 0.05, *p* < 0.01, *p* < 0.001; Figure 1A,B). During follicle development from WF to small yellow follicles (SYF), *VDR* mRNA transcript abundance increased but *PDIA3* decreased. In the granulosa layer of yellow preovulatory follicles, transcript levels of both receptors decreased along with transition from F3 to F1 (Figure 1A,B).

The levels of *CYP27C1* and *CYP24A1* mRNA transcripts differ between ovarian tissues (*p* < 0.05, *p* < 0.01, *p* < 0.001). The highest abundances of these enzyme mRNA transcripts were in the WF, and lower in more developed follicles (Figure 1C,D). In the granulosa and theca layers of the largest preovulatory follicles, *CYP24A1* mRNA transcript abundances increased with progress in follicle maturation (Figure 1D).

In additionally examined tissues, *VDR* mRNA transcript abundances were higher in the kidney and shell gland than in the pituitary and liver (*p* < 0.05, *p* < 0.001; Figure 2A). *PDIA3* mRNA levels were similar in the pituitary, liver, kidney, and shell gland (*p* > 0.05; Figure 2B). *CYP27C1* mRNA transcript level was the highest in the pituitary, lower in the shell gland and kidney, and the lowest in the liver (*p* < 0.05, *p* < 0.01, *p* < 0.001; Figure 2C). *CYP24A1* mRNA transcript abundance was the highest in the kidney, followed by the pituitary, shell gland, and liver (*p* < 0.01, *p* < 0.001; Figure 2D).

### 2.2. Abundances of VDR, PDIA3, 1α-hydroxylase, and 24-hydroxylase Protein in the Hen Reproductive System

**Figure 3:**
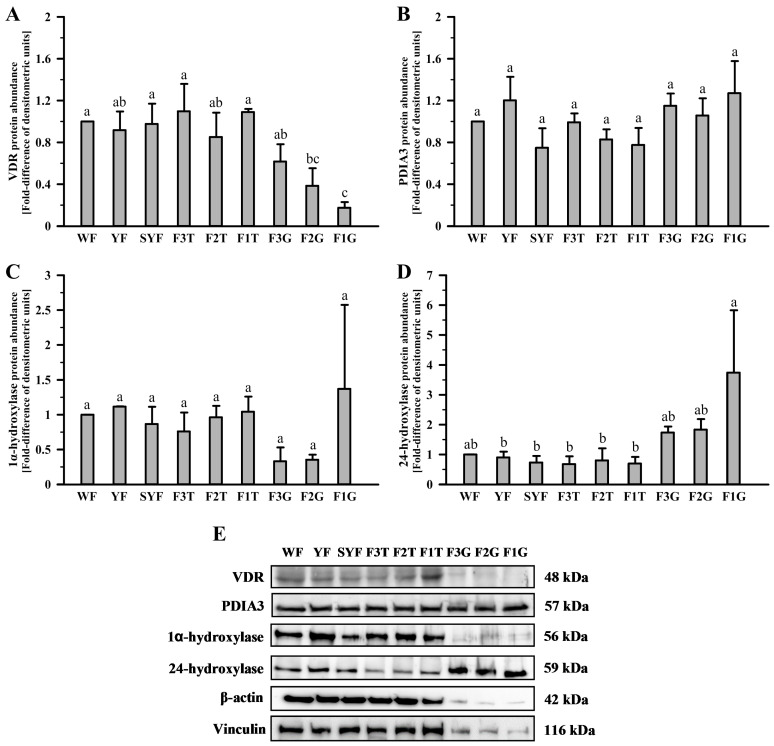
Western blot analysis of VDR (**A**,**E**), PDIA3 (**B**,**E**), 1α-hydroxylase (**C**,**E**), and 24-hydroxylase (**D**,**E**) protein in the hen ovary. Graphs depict the relative protein abundances normalized to the vinculin protein. Data are expressed as fold difference ± standard error of the mean, using tissues from three different biological replicates (hens), and are compared to the abundance in the WF, considered to be 1. Values marked with different letters differ significantly (*p* < 0.05; Kruskal–Wallis ANOVA on ranks, followed by the Student–Newman–Keuls test). Images (**E**) are representative of three independent blots. Abbreviations as in Figure 1.

**Figure 4:**
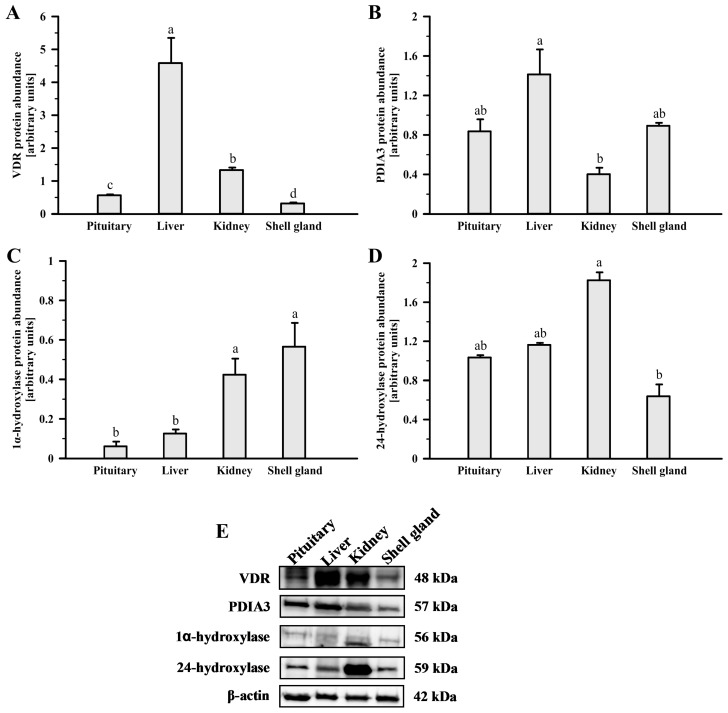
Western blot analysis of VDR (**A**,**E**), PDIA3 (**B**,**E**), 1α-hydroxylase (**C**,**E**), and 24-hydroxylase (**D**,**E**) protein in the hen pituitary, liver, kidney, and shell gland. Graphs depict the relative protein abundances normalized to β-actin protein. Data are expressed as the mean relative abundance ± standard error of the mean, using tissues from three different biological replicates (hens). Values marked with different letters differ significantly (*p* < 0.05; Kruskal–Wallis ANOVA on ranks, followed by the Student–Newman–Keuls test). Images (**E**) are representative of three independent blots. Abbreviations as in Figure 1.

The abundances of VDR, PDIA3, 1α-hydroxylase, and 24-hydroxylase protein in the hen ovary, pituitary, liver, kidney, and oviductal shell gland were examined by a Western blot analysis (Figure 3 and Figure 4). Antibodies recognized bands with predicted molecular weights of 48, 57, 56, and 59 kDa, respectively (Figure 3 and Figure 4, bottom panels).

In the ovary, the abundance of VDR protein was lower in the granulosa layer of F2 and F1 follicles compared to theca layers of F3-F1 follicles and less mature follicles (*p* < 0.05, *p* < 0.01, *p* < 0.001). PDIA3 and 1α-hydroxylase protein abundances were similar in all ovarian tissues (Figure 3B,C). 24-hydroxylase protein abundance was the highest in the granulosa layer of the F1 follicle (*p* < 0.05, *p* < 0.01, *p* < 0.001). There were no differences in 24-hydroxylase protein abundances among other ovarian tissues (Figure 3D).

Among the additional examined tissues, VDR protein abundance was the highest in the liver, followed by the kidney, pituitary, and shell gland (*p* < 0.05; Figure 4A). PDIA3 protein level was lower in the kidney than in the liver (*p* = 0.017) and did not differ between the liver, pituitary, and shell gland (*p* > 0.05; Figure 4B). Abundances of 1α-hydroxylase protein were higher in the shell gland and kidney than in the liver and pituitary (*p* < 0.05, *p* < 0.01, *p* < 0.001; Figure 4C). 24-hydroxylase protein abundance was higher in the kidney than in the shell gland (*p* = 0.024) and did not differ between the pituitary, liver, and kidney (*p* > 0.05; Figure 4D).

### 2.3. Immunofluorescent Localization of PDIA3, 1α-hydroxylase, and 24-hydroxylase in the Hen Reproductive System

**Figure 5:**
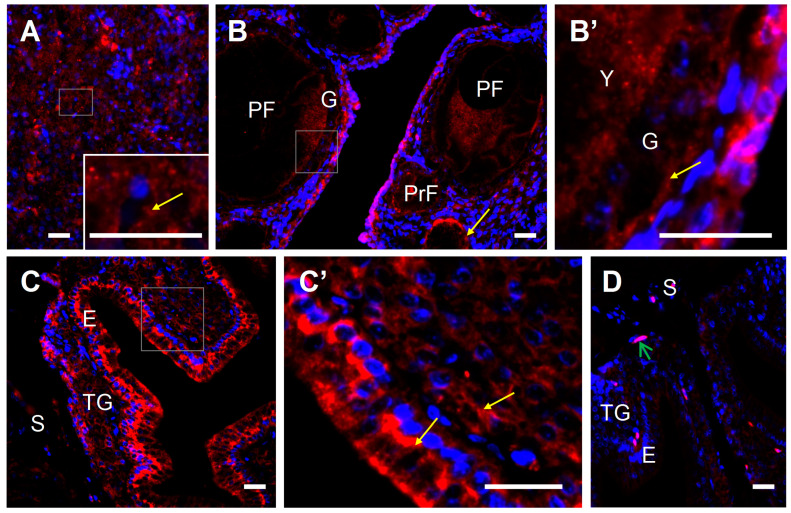
Representative micrographs of PDIA3 protein immunofluorescent localization in the laying hen liver (**A**), ovary (**B**,**B’**), and shell gland (**C**,**C’**). Immunoreactivity was visualized using a DyLight 594 system. Cell nuclei were counterstained with DAPI (blue fluorescence). Positive signals (red fluorescence) for PDIA3 (yellow arrows) were found in the cytoplasm of liver cells (**A**), granulosa cells of primordial follicles (composed of one layer of granulosa cells) and primary follicles (composed of one layer of granulosa cells and a thin theca layer) located in the ovarian stroma (**B**,**B’**), and mainly basal cells of the luminal epithelium and tubular gland cells of the shell gland (**C**,**C’**). A representative negative control section (shell gland) incubated without primary antibody does not exhibit positive staining (**D**), excluding red blood cells which always show nonspecific fluorescence (green arrow). Frames indicate the location of the higher magnification view (insert in (**A**); photos (**B’**) and (**C’**)). Abbreviations: PrF, primordial follicles; PF, primary follicle; G, granulosa; Y, yolk; E, luminal epithelium; TG, tubular glands; S, stroma (loose connective tissue + muscles). Scale bars = 20 µm.

Positive red immunofluorescence for PDIA3 protein was localized in cells of all examined tissues, including the granulosa and theca layers of the wall of ovarian follicles, pituitary, and luminal epithelium and tubular glands of the shell gland (Figure 5).

**Figure 6:**
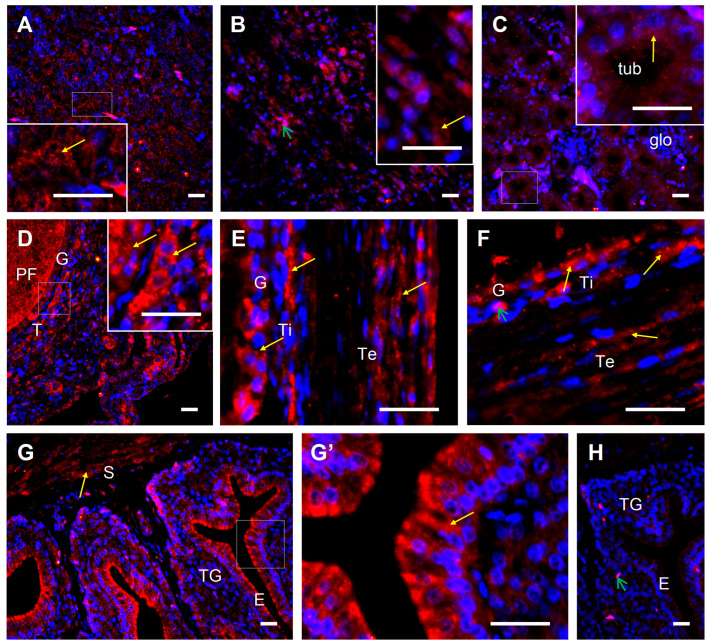
Representative micrographs of 1α-hydroxylase protein immunofluorescent localization in the laying hen pituitary (**A**), liver (**B**), kidney (**C**), ovary (**D**–**F**), and oviductal shell gland (**G**,**G’**). Immunoreactivity was visualized using a DyLight 594 system. Cell nuclei were counterstained with DAPI (blue fluorescence). Positive signals for 1α-hydroxylase (red fluorescence, yellow arrows) were found in the cytoplasm of pituitary cells (**A**), liver cells (**B**), tubule cells in the kidney (**C**), and ovarian stroma cells (**D**), as well as the granulosa and theca cells of primary follicles ((**D**); composed of one layer of granulosa cells and a thin theca layer), yellowish follicles ((**E**); 4–8 mm in diameter; composed of several layers of granulosa cells, theca interna and externa, and epithelium with loose connective tissue), and yellow follicles ((**F**); > 8 mm; composed of one layer of granulosa cells, theca interna and externa, and epithelium with loose connective tissue). Moreover, immunopositive signals were observed in the shell gland (**G**,**G’**). Strong intensity of staining is noted in apical cells of the luminal epithelium, moderate or weak in tubular glands, and moderate in muscle cells of the stroma. A representative negative control section (shell gland) incubated without primary antibody does not exhibit positive staining (**H**), excluding red blood cells, which always show nonspecific fluorescence (green arrows). Frames indicate the location of the higher magnification view (insert in (**A**–**D**), photo (**G’**)). Abbreviations: glo, glomerulus; tub, tubule; T, theca; Ti, theca interna; Te, theca externa. Scale bars = 20 µm. Other abbreviations and explanations as in Figure 5.

The 1α-hydroxylase protein was found in the wall of ovarian follicles at different stages of development, pituitary, liver, kidney, and oviductal shell gland (Figure 6). Within the follicular wall, a strong immunopositive reaction for 1α-hydroxylase was present in the cytoplasm of granulosa cells and both theca interna and externa layer cells (Figure 6D–F). Within the wall of the shell gland, strong positive signals were observed in the luminal epithelium, and less intense in tubular glands and muscles located in the stroma (Figure 6G,G’).

**Figure 7:**
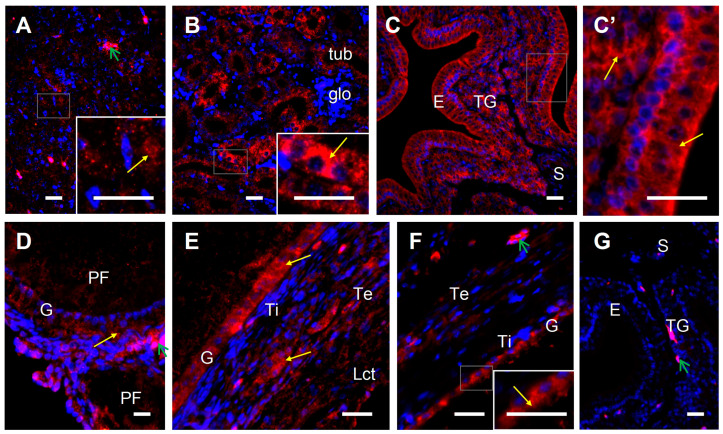
Representative micrographs of 24-hydroxylase protein immunofluorescent localization in the laying hen pituitary (**A**), kidney (**B**), shell gland (**C**,**C’**), and ovary (**D**–**F**). Immunoreactivity was visualized using a DyLight 594 system. Cell nuclei were counterstained with DAPI (blue fluorescence). Positive signals for 24-hydroxylase (red fluorescence, yellow arrows) were found in the cytoplasm of pituitary cells (**A**), tubule cells in the kidney (**B**), and luminal epithelium and tubular gland cells of the shell gland (**C**,**C’**), as well as the granulosa and theca cells of primary follicles (**D**), white follicles ((**E**); 1–4 mm in diameter; composed of several layers of granulosa cells, theca interna and externa, and epithelium with loose connective tissue), and yellow follicles ((**F**); > 8 mm). A representative negative control section (shell gland) incubated without primary antibody does not exhibit positive staining (**G**), excluding red blood cells which always show nonspecific fluorescence (green arrows). Frames indicate the location of the higher magnification view (insert in (**A**–**C,F**); photo (**C**’)). Abbreviation: Lct, epithelium with loose connective tissue. Scale bars = 20 µm. Other abbreviations and explanations as in Figure 5 and Figure 6.

The 24-hydroxylase protein was localized in cells of all investigated tissues, i.e., ovarian follicles, pituitary, kidney, and shell gland (Figure 7), confirming the results from the Western blot analysis. Within the follicular wall, a positive immunoreactivity was observed primarily in the cytoplasm of granulosa cells. In the wall of the shell gland, positive signals were present in cells of the luminal epithelium and tubular glands.

There was no red immunofluorescence when sections were incubated with normal rabbit serum (Figure 5D, Figure 6H and Figure 7G) instead of a primary antibody.

## 3. Discussion

Previously, it has been shown that the chicken ovary (mainly the granulosa cells) and oviduct as well as some other organs such as the kidney, express VDR; therefore, there are target tissues for vitamin D_3_ genomic action [23,24,25,26]. In the present study, besides VDR, we demonstrated, for the first time, the mRNA and protein abundance, and protein localization of another vitamin D_3_ receptor (PDIA3), and hydroxylases (*CYP27C1* and *CYP24A1*) responsible for vitamin D_3_ metabolism in the chicken ovarian follicles, oviductal shell gland, pituitary, liver, and kidney. Although we were not able to detect VDR protein immunoreactivity in examined tissues with an applied antibody, the presence of a *VDR* transcript and protein were confirmed in the ovarian and oviductal tissues. Generally, the results obtained herein are in line with previous studies [24,25,26], and additionally, clearly show the expression of VDR in the theca layer of ovarian follicles and pituitary, indicating a genomic action of vitamin D_3_ also in these tissues. There were discrepancies between the mRNA and protein abundances in some tissues such as the granulosa layer of F3 to F1 follicles, liver, and kidney, where mRNA abundances were higher but protein abundances were lower compared to other tissues. It may be attributable, at least in part, to posttranscriptional regulatory mechanisms.

Of particular interest in this study was the finding of *PDIA3* mRNA transcript and protein in all examined tissues, which is the first evidence of potential rapid effect of vitamin D_3_ in the chicken reproductive tissues. In detail, *PDIA3* mRNA abundances differed between ovarian follicles/tissues and were the lowest in the theca layer of F3-F1 follicles, but there were no differences in PDIA3 protein abundances. Within the follicular wall, PDIA3 protein was localized in both the granulosa and theca layers. These results indicate a rapid non-genomic action of vitamin D_3_ in the chicken ovarian follicles similar to porcine follicles [22]. Furthermore, *PDIA3* mRNA transcript levels were comparable between tissues, and protein levels were higher in the liver, pituitary, and shell gland than in the kidney. Presence of PDIA3 protein detected by Western blot in these organ cells was also confirmed by immunofluorescence. More specifically, in the shell gland, PDIA3 was immunolocalized in cells of the luminal epithelium and tubular glands. These observations suggest that the kidney and all organs associated with reproduction (ovary, mainly the mucosa of shell gland, pituitary, and liver) are target tissues for biological action of vitamin D_3_, mediated by not only the genomic with participation of VDR, but also non-genomic pathways with involvement of PDIA3. It should be noted that VDR can also work as a membrane receptor for vitamin D_3_ [20]. Moreover, in all compartments of the hen ovary, the expression of the retinoid X receptor (RXR) occurs [29]. VDR forming a VDR-RXR heterodimer may cooperate with RXR in signal transduction initiated by vitamin D_3_ as well. Further studies are necessary to examine VDR and PDIA3 expression according to the stage of the ovulatory and egg formation cycle as well as specific roles of vitamin D_3_ in particular tissues/organs in birds.

In the ovary, vitamin D_3_ by its receptors may have a role in the regulation of steroidogenesis process. This is suggested by decreased circulating concentrations of estradiol and progesterone in hens maintained on a vitamin D_3_-deficient diet [27]. Data from mammalian studies have also indicated the importance of vitamin D_3_ in the regulation of ovarian steroidogenesis. For example, vitamin D_3_ increased in vitro secretion of estradiol by small and medium antral follicles, but not by large ones in pigs [22], as well as increased secretion of progesterone by granulosa cells in goats via the upregulation of *StAR* and *3β-HSD* genes [30]. Further studies are necessary to explain the involvement of vitamin D_3_ in the ovarian steroidogenesis in birds. Another role of vitamin D_3_ in the ovary may potentially be attributable to the regulation of follicle selection into preovulatory stage of follicle development. So far, it has been revealed that in vitro vitamin D_3_ stimulates proliferation of granulosa cells isolated from chicken prerecruited follicles [24], elevates mRNA transcript abundances of follicle stimulating hormone (FSH) receptor and *Kit ligand*, but decreases anti-Mullerian hormone (AMH) transcript levels in these follicles [24,28]. It is likely that vitamin D_3_ can regulate the expression of other genes and/or modulate different cell signaling pathways determining the follicle fate in the hen ovary.

It is worth mentioning that the shell gland (uterus) is an oviductal segment where about 2–2.5 g of calcium is deposited in the eggshell of a single egg [31]. On the other hand, vitamin D_3_ plays a crucial role in the regulation of calcium metabolism [31,32] mediated through VDR and the role of vitamin D_3_ is more important in the shell gland than in other oviductal parts [25,26]. As was previously shown, the expression of calbindin D28K (calcium binding and intracellular transporting protein) in the shell gland is under the direct regulation of vitamin D_3_ [33]. It cannot be excluded that other molecules contributing to the eggshell formation are under control of vitamin D_3_, mediated by both VDR and PDIA3, which are plentiful in the luminal epithelium and tubular glands of the shell gland ([26], present study).

The next novel finding of this study was the detection of vitamin D_3_ metabolizing enzymes in chicken ovarian follicles along with their development. Within the wall of follicles, 1α-hydroxylase and 24-hydroxylase were localized in the granulosa and theca cells. Although the profiles of *CYP27C1* and *CYP24A1* mRNA transcript abundances did not exactly match the protein abundance profiles, it seems that the smaller follicles are metabolic sites of vitamin D_3_ in larger part, compared to preovulatory follicles. Moreover, in large yellow follicles, mostly the theca cells, synthesize biologically active vitamin D_3_, and granulosa cells inactivate this vitamin. These observations, provided for the first time, indicate that chicken ovarian follicles can metabolize vitamin D_3_ and that vitamin D_3_ may be an autocrine/paracrine regulator of follicle development. Previously, follicle size-dependent expression of 1α-hydroxylase and 24-hydroxylase, as well as their localization in the granulosa and theca cells were demonstrated in the pig ovary [22].

In a further step of our study, we demonstrated 1α-hydroxylase and 24-hydroxylase in the shell gland in addition to the kidney, liver, and pituitary gland. Interestingly, mRNA transcript and protein abundances of 1α-hydroxylase (*CYP27C1*) in the shell gland were comparable, whereas 24-hydroxylase (*CYP24A1*) levels were lower than in the kidney, known as a main organ of active vitamin D_3_ metabolism [13,14]. These results prompt us to assume that the shell gland of hens is another important organ of the active form of vitamin D_3_ synthesis, and also an extra-renal site of vitamin D_3_ inactivation. The 1α-hydroxylase was localized primarily to the luminal epithelium and muscles of the shell gland, and 24-hydroxylase to the luminal epithelium and tubular glands. Our results regarding detection of vitamin D_3_ metabolizing enzymes, closely correspond to those in the pig [16] and sheep [34] uterus. Above observations may further suggest that locally produced vitamin D_3_ is involved in the control of synthesis/action of different proteins associated with the eggshell formation in epithelial and tubular gland cells, as well as calcium metabolism related to muscle contractility. Inactivation of vitamin D_3_ in the oviductal shell gland seems to take place mainly in the luminal epithelium and tubular glands.

## 4. Materials and Methods

### 4.1. Chickens and Tissue Sampling

Hy-Line Brown laying hens (egg layer) were caged under temperatures of about 16 °C and a photoperiod of 14L:10D, with free access to feed and water. At the age of 28 weeks, hens (*n* = 9) were humanely euthanized about 2 h after the oviposition when they contained an egg in the magnum and the following ovarian compartments were harvested: white follicles (WF), yellowish follicles (YF), small yellow follicles (SYF), and the theca (T) and granulosa (G) layers of three of the largest yellow preovulatory follicles F3-F1 (F3 < F2 < F1). Additionally, the pituitary, liver, kidney, and oviductal shell gland were collected. Tissue samples (*n* = 6 hens) were divided into two parts, immediately frozen in liquid nitrogen and kept at −80 °C or placed into RNAlater (Sigma-Aldrich, Saint Louis, MO, USA) for future determination of protein expression or RNA isolation, respectively. The other tissues (*n* = 3 hens) were fixed in freshly prepared 10% (v:v) buffered (0.1 M phosphate buffer, pH 7.6) formalin, processed, and embedded in paraffin wax for the subsequent immunofluorescence analysis.

### 4.2. RNA Isolation and qRT-PCR

Total RNA isolation, reverse transcription, and quantitative real-time polymerase chain reaction (qRT-PCR) were performed as previously described [35]. According to the manufacturer’s protocol, total RNA was isolated from tissue samples with TRI-reagent (Sigma-Aldrich), and complementary DNA (cDNA) was synthesized using a High-Capacity cDNA Reverse Transcription Kit (Applied Biosystems, Foster City, CA, USA). The relative abundance of mRNA encoding *VDR*, *PDIA3*, *CYP24A1*, and *CYP27C1* was determined using the chicken-specific TaqMan Gene Expression Assays (Table 1; Applied Biosystems) in a StepOne Plus thermocycler (Applied Biosystems).

qRT-PCR was performed with the following conditions: 50 °C for 2 min and 95 °C for 10 min, followed by 40 cycles at 95 °C for 15 s and 60 °C for 60 s. The duplex qRT-PCR for the examined gene and *18S rRNA* (reference gene) was performed (each sample in duplicate) in a volume of 10 μL, containing 5 μL TaqMan Gene Expression Master Mix (Applied Biosystems), 0.5 μL TaqMan Gene Expression Assay with specific pair of primers and TaqMan MGB-probe designed by Applied Biosystems, 0.5 μL of Eukaryotic *18S rRNA* Endogenous Control (Table 1), 3 μL water, and 1 μL cDNA (10× diluted after the reverse transcription). A no-template control was included in each run. Relative quantification of the examined genes was calculated after normalization with the *18S rRNA* transcript, and expression in the WF (when compared between ovarian tissues; Figure 1) or pituitary (when compared between non ovarian tissues; Figure 2) as the calibrator, by using the 2^−ΔΔCt^ method.

### 4.3. Western Blot Analysis

Tissue samples were homogenized in a lysis buffer (BioVision, Milpitas, CA, USA), sonicated, and centrifuged at 4°C for 20 min at 10,000× *g*. The protein concentration was estimated by Bradford protein assay (Bio-Rad, Hercules, CA, USA) with bovine serum albumin (BSA) as the standard. Subsequent Western blot analysis were performed as per a previously described protocol [36]. Samples were separated by 12% SDS–PAGE (Mini-Protean TGX Precast Gels; Bio-Rad Laboratories Inc., GmbH, Munich, Germany) and electroblotted onto a PVDF membrane (Trans-Blot Turbo Mini 0.2 µm PVDF Transfer Packs; Bio-Rad Laboratories Inc.) using a semi–dry Trans-Blot Turbo Transfer System (Bio-Rad Laboratories Inc.). The blotted membranes were blocked for 1 h at room temperature in 5% non-fat dry milk containing 0.1% Tween20 followed by overnight incubation at 4 °C with primary antibodies and then with a secondary horseradish peroxidase-conjugated antibody for 1.5 h at room temperature (Table 2). Proteins were detected by chemiluminescence and images were captured with a ChemiDocTM XRS+ System (Bio-Rad Laboratories Inc.). Each membrane was stripped and reprobed with an anti-β-actin antibody followed by a respective secondary antibody (Table 2). Additionally, samples from ovarian follicles (WF, YF, SYF, F3T-F1T, F3G-F1G) were reprobed with vinculin as another endogenous control. The bands were densitometrically quantified and normalized to their corresponding β-actin or vinculin (for ovarian follicle samples) bands using the public domain ImageJ program v. 1.8.0 (National Institutes of Health, Bethesda, MD, USA). Results from YF, SYF, F3T-F1T and F3G-F1G samples were expressed as a fold-difference compared to level in the WF set as 1.

### 4.4. Immunofluorescence

The immunofluorescence assays for VDR, PDIA3, 24-hydroxylase, and CYP27B1 protein were performed routinely as described previously [16,35]. Briefly, after deparaffinization and rehydration, tissue sections were microwaved in 0.01M citric buffer (pH 6.0) to epitope retrieval. Non-specific binding sites were blocked with 5% normal goat serum for 20 min. Sections were then incubated overnight (4 °C) in the presence of primary antibodies (Table 2), followed by incubation with secondary antibodies (Table 2) and mounted with VECTASHIELD^®^ Vibrance™ Antifade Mounting Medium with DAPI (Vector Laboratories, Burlingame, CA, USA). Non-specific staining was verified by the substitution of the primary antibody with normal rabbit serum. Sections were examined with an Axio Scope.A1 fluorescent microscope and photographed with an Axiocam 503 color camera and the ZEN 2.3 pro software (Carl Zeiss, Jena, Germany). The intensity of the immunoreactivity was estimated as strong, moderate, weak, and very weak. Micrographs show a merge of blue fluorescence representing DAPI staining of cell nuclei and red fluorescence representing immunopositive reaction specific for examined protein.

### 4.5. Statistical Analysis

To verify the distribution of data, the Shapiro–Wilk test was applied. Homogeneity of variance was assessed with the Brown–Forsythe test. Data of relative gene expression and relative abundance of proteins were statistically analyzed by the nonparametric Kruskal–Wallis ANOVA on ranks, followed by the Student–Newman–Keuls test. Differences in values were considered significant at the 95% confidence level (*p* < 0.05). Calculations were performed with SigmaPlot_V_13 (Systat Software Inc., San Jose, CA, USA). Results are presented as the arithmetic mean ± standard error of the mean (SEM).

## 5. Conclusions

Collectively, this study, for the first time, has demonstrated by means of qRT-PCR, Western blot, and immunohistochemistry, that ovarian follicles and the oviductal shell gland of the hen possess two kind of vitamin D_3_ receptors. In addition to VDR primarily involved in genomic action, there is PDIA3 likely participating in rapid, non-genomic influence of vitamin D_3_. Furthermore, the finding of 1α-hydroxylase (*CYP27C1*) and 24-hydroxylase (*CYP24A1*) expression in reproductive tissues indicates that the reproductive system in hens have a potential for vitamin D_3_ synthesis and inactivation and may suggest that locally produced vitamin D_3_ can be considered as an important regulator of ovarian and shell gland function in birds. These results provide a new insight into the potential mechanisms of vitamin D_3_ action and metabolism in the chicken ovary and oviduct. Understanding of vitamin D_3_ role in molecular mechanisms underlying functioning of the reproductive system and formation of high quality eggs in hens may be of considerable importance for poultry production.

## Figures and Tables

**Table 1:** GenBank identifications, assay ID, and amplicon lengths generated by a quantitative real-time polymerase chain reaction (qRT-PCR) assay for chicken vitamin D_3_ receptors- and metabolism-related genes.

Gene	GeneBank Identification	Assay ID	Amplicon Size [bp]
*VDR*	NM_205098.1	Gg03348521_g1	80
*PDIA3*	NM_204110.3	Gg03346843_m1	90
*CYP27C1*	XM_004942891.3	Gg03326395_m1	107
*CYP24A1*	AF019142.1	Gg03347931_m1	90
*18S rRNA*	X03205.1	Cat. no. 4310893E	187

Assay-on-Demand, TaqMan MGB Gene Expression Kits were used for analysis of all gene mRNA expression. Abbreviations: *CYP24A1*, cytochrome P450 family 24 subfamily A member 1 = 24-hydroxylase; *CYP27C1*, cytochrome P450 family 27 subfamily C member 1 = 1α-hydroxylase; *PDIA3*, protein disulfide isomerase family A member 3; *VDR*, vitamin D_3_ (1α,25- dihydroxyvitamin D_3_) receptor.

**Table 2:** Primary and secondary antibodies used for Western blot (WB) and immunofluorescence (IF).

Antibody	Serum	Host Species	Vendor	Cat. No	WB Dilution	IF Dilution
Anti-VDR	5% NGS	Rabbit	Cell Signaling, Massachusetts, DA, USA	12550	1:1000	1:50
Anti-PDIA3	5% NGS	Rabbit	Proteintech, Manchester, UK	15967-1-AP	1:5000	1:200
1α-hydroxylase	5% NGS	Rabbit	Invitrogen, Carsband, CA, USA	PA5-79128	1:2000	1:200
24-hydroxylase	5% NGS	Rabbit	Invitrogen, Carsband, CA, USA	PA5-79127	1:1000	1:150
Anti-β-actin	-	Mouse	Sigma-Aldrich, St. Louis, MO, USA	A2228	1:4000	-
Anti-vinculin	-	Mouse	Sigma-Aldrich, St. Louis, MO, USA	V9264	1:2000	-
HRP-anti mouse IgG	-	Horse	Bio-Rad Laboratories Inc., GmbH, Munich, Germany	170-6516	1:3000	-
HRP-anti rabbit IgG	-	Goat	Invitrogen, Carsband, CA, USA	31460	1:3000	-
DyLight 594-anti rabbit	5% NGS	Goat	Vector Laboratories, Burlingame, CA, USA	DI-1594-1.5	-	1:150

Abbreviations: HRP, horseradish peroxidase; NGS, normal goat serum; NHS, normal horse serum; PDIA3, protein disulphide isomerase family A, member 3; VDR, vitamin D receptor.

## Data Availability

The raw data used for the preparation of the presented results are available on request from the corresponding authors.
